# Short report: The potential of PCR on skin flakes from bed linens for diagnosis of scabies in an outbreak

**DOI:** 10.1371/journal.pntd.0009485

**Published:** 2021-06-14

**Authors:** Raïssa Tjon-Kon-Fat, Laurène Peckeu, Susan Hahné, Eric de Coster, Wendy Tas, Bas Wintermans, Anneke Bergmans, Mariska Petrignani, Ewout Fanoy

**Affiliations:** 1 European Programme for Intervention Epidemiology Training (EPIET), European Centre for Disease Prevention and Control (ECDC), Stockholm, Sweden; 2 Centre for Infectious Disease Control Netherlands (CIb), National Institute for Public Health and the Environment (RIVM), Bilthoven, the Netherlands; 3 Public Health Service of the region of The Hague (GGD Haaglanden), The Hague, the Netherlands; 4 Microvida, location Bravis, Roosendaal, the Netherlands; 5 Public Health Service of the region of Amsterdam (GGD Amsterdam), Amsterdam, the Netherlands; 6 Public Health Service of the region of Rotterdam-Rijnmond (GGD Rotterdam-Rijnmond), Rotterdam, the Netherlands; Washington University School of Medicine, UNITED STATES

## Abstract

**Background:**

Scabies outbreaks are common in nursing homes in the Netherlands. In October 2018, a local public health service (PHS) in The Hague was notified of a new scabies outbreak in a nursing home in that region. The PHS initiated an outbreak investigation. Cases were defined as: possible (reported symptoms), probable (scabies-like lesions) and confirmed (PCR or microscopy in skin flakes). Head-to-toe examinations were performed of all residents and those staff members who reported symptoms suggestive of scabies. Skin scrapings of lesions were tested either with microscopy or by PCR. Experimentally for case finding, skin flakes from bed linens of residents who reported symptoms of itchiness but did not have primary lesions were sent for PCR testing.

**Principal findings:**

All residents (41) and 37/44 staff were included in this outbreak investigation. We identified 30 possible, four probable and six confirmed cases. The overall attack rate for probable/confirmed cases was 10/78 (13%). Of the six confirmed cases, two were confirmed by PCR, three by microscopy, and one showed positive findings with both techniques. Two out of the three bed-linen specimens were PCR-positive.

**Conclusions:**

In this outbreak of scabies in a nursing home, PCR was used on skin flakes from bed linens, which led to the detection of two additional cases. This illustrates the potential of PCR during the investigation of scabies outbreaks.

## Author summary

Scabies is a very common skin condition that is caused by tiny mites that lay eggs in the skin. One of the first symptoms that are reported by persons who are infested with scabies is intense itching, especially at night. Typically a rash occurs which affects certain parts of the body, for instance between the fingers. Without treatment scabies could lead to secondary skin infections caused by bacteria due to persistent scratching. Outbreaks of scabies tend to occur in settings where people live in close quarters, such as nursing homes. In October 2018 there was an outbreak of scabies in a nursing home in the region of The Hague in the Netherlands. Of the 78 staff members and residents included in the investigation, ten persons had typical lesions or tested positive for scabies using PCR and/or microscopy on skin samples. For two persons who tested positive for scabies, PCR test was done on skin samples from bed linens. Testing of skin samples from bed linens is a technique not previously used for the diagnosis of scabies prior to the current investigation. This illustrates the potential of using this technique to help diagnose scabies during outbreaks.

## Introduction

Human scabies is a skin infestation caused by the mite *Sarcoptes scabiei var*. *hominis*. The mite can burrow into the epidermis of the skin when an infested person has close contact with a new host. The classical clinical presentation of scabies is intense itching and erythematous papules and/or burrows [1].

Scabies is one of the most common skin diseases in developing countries [1]. In high-income countries, scabies outbreaks are seen mostly in care facilities, specifically nursing homes [2]. In these settings timely outbreak detection is crucial to implement appropriate control measures. Diagnosis through laboratory techniques is recommended, as the clinical presentation might differ in the elderly and control measures are labor intensive and have a huge social and financial impact [2]. Contact tracing around a confirmed case can identify additional scabies cases, which is necessary to assess the size of the outbreak and determine the contacts eligible for preventive treatment.

Definitive diagnosis of scabies is made by the identification of mites, eggs or other mite products like excreta, usually performed visually by dermatoscopy or microscopy [3]. Microscopy is known to be a highly specific diagnostic technique, but it has a low sensitivity when low numbers of mites are present. Therefore diagnosis of scabies remains difficult, as a negative result does not exclude scabies. Polymerase chain reaction (PCR) has been suggested to be a more sensitive technique to diagnose scabies in those patients with a subtle clinical presentation [4]. The PCR test can detect the presence of cellular DNA from mites, eggs, and excreta deposed on or in the human skin of an infested person. Previous molecular studies of scabies have been published that describe various real-time and conventional PCRs [4–5]. A real-time PCR targeting the ITS2 region of *S*. *scabiei* DNA was previously developed [6]. Amplification was performed using the primers Scab-F1 (5’- ATGTGTGCCTGTTGAGATTGAGA -3’), Scab-R1 (5’-CTGAGGTCGAGAAATGACATTTCA-3’) and probe Scab-FAM-MGB (5’-FAM-CAAACATGAATATCAAAGAGTGAT-MGB-3’). In a validation study on 77 skin samples from potential scabies cases, the PCR showed a sensitivity of 87% (compared to a sensitivity of 77% for microscopy) and a specificity of 100% [6].

In the context of using PCR during scabies outbreaks, a recent study showed that this assay was highly specific with similar sensitivity compared to microscopy, and was easy to use for health care workers with no scabies diagnostic expertise [7]. Moreover, another study showed similar results and recommended PCR as a diagnostic tool in scabies outbreaks [8].

In a recent outbreak of scabies in a nursing home in the Netherlands, the regional Public Health Service (PHS) used PCR and microscopy on skin flakes from skin lesions. Moreover, experimental PCR testing was performed on skin flakes from bed linens in order to detect additional cases of scabies. In this short report, we describe the findings of this outbreak investigation.

## Methods

### Ethics statement

Outbreaks of scabies should be notified to the Public Health Service as described under the Public Health Act, and therefore do not require separate medical ethical clearance. The PHS developed an informed consent form for staff and residents asking for permission to use their personal and medical information for this outbreak investigation. This form was distributed to the staff by the management of the nursing home. For the residents, permission was obtained through the family representative.

### Outbreak investigation

On 16 October 2018, the PHS of The Hague was alerted to a probable scabies outbreak in a nursing home that houses residents who either have a chronic psychiatric condition in combination with a physical disability or a chronic psychiatric condition and need a temporary nursing home for rehabilitation. In total approximately 41 residents live at the two affected units of the nursing home, who are being taken care for by around 44 staff members. Five previous outbreaks of scabies had occurred in this nursing home in the past four years.

On 19 October 2018, the doctors and nurses of the PHS visited the nursing home. The PHS conducted interviews with residents and obtained data on their medical history from the medical records. Due to the residents’ psychiatric conditions, the PHS felt that residents might not be able to express whether they had symptoms, and therefore physical examination was planned to be performed on all residents, regardless of having reported symptoms suggestive of scabies. If these residents had any lesions that could be suggestive of scabies during the physical examination, skin scrapings were taken for laboratory confirmation. These specimens were first tested using microscopy. In case these were negative, left over material or consecutive samples were sent to the laboratory for PCR testing. Experimentally for residents who reported symptoms of itchiness but had no primary lesions indicative of scabies, bed linens were investigated for the presence of skin flakes. Specimens were collected by scotch tape or in a urine container and tested by microscopy and subsequently by PCR.

Furthermore, on this day the management of the nursing home had asked those staff members that had reported itching or other symptoms suggestive of scabies to be present at the nursing home. These staff members also underwent a head to toe physical examination, and skin scrapings were taken for laboratory confirmation (PCR and/or microscopy) following the same procedures as described above for residents.

### Case definition

Three categories of cases were defined as followed (adapted from [2]):

- Confirmed case: a resident or staff member of this nursing home, who presented with clinical features suggestive of scabies (i.e. primary or secondary lesions) plus a positive confirmation with either microscopy or PCR
- Probable case: a resident or staff member of this nursing home, who presented with clinical features suggestive of scabies (i.e. primary or secondary lesions) with a negative test result with either microscopy, PCR or both methods
- Possible case: a resident or staff member of this nursing home, who reported symptoms of itchiness but had no clinical features suggestive of scabies (i.e. primary or secondary lesions) and no laboratory test performed or negative with either microscopy, PCR or both methods

Primary lesions are defined as burrows, papules, nodules, or vesicles; secondary lesions are excoriations due to scratching.

## Results

This outbreak occurred among 85 residents and staff members of a nursing home. 78 (92%) participated in the outbreak investigation: all 41 residents and 37 out of the 44 staff members. Seven staff members did not participate due to holidays, longer absences from work or no response. Of the 78 persons included in this outbreak investigation, 30 persons met the possible case definition (i.e. reporting symptoms of itchiness), four persons met the probable case definition (i.e. signs suggestive of scabies without laboratory confirmation) and six met the confirmed case definition (i.e. symptoms suggestive of scabies with laboratory confirmation with PCR and/or microscopy) (**Table 1**). 36 of the 41 residents and 9 out of 37 staff members were physically examined. We did not diagnose any cases of crusted scabies among residents nor staff members during this outbreak.

**Table 1:** The number of cases for residents and staff.

	Residents N = 41	Staff N = 37
Confirmed	5	1
Probable	3	1
Possible	17	13
**Total number of cases**	**25**	**15**
Attack rate (%) for all cases	61	41
Attack rate (%)–probable and confirmed cases	20	5.4

The overall attack rate was 51% when including all cases, and 13% when only including probable and confirmed cases. The majority (63%) of the cases were residents. The attack rate was higher among residents (20%) compared to that for staff members (5.4%) when only including probable and confirmed cases (**Table 1**).

**Fig 1:**
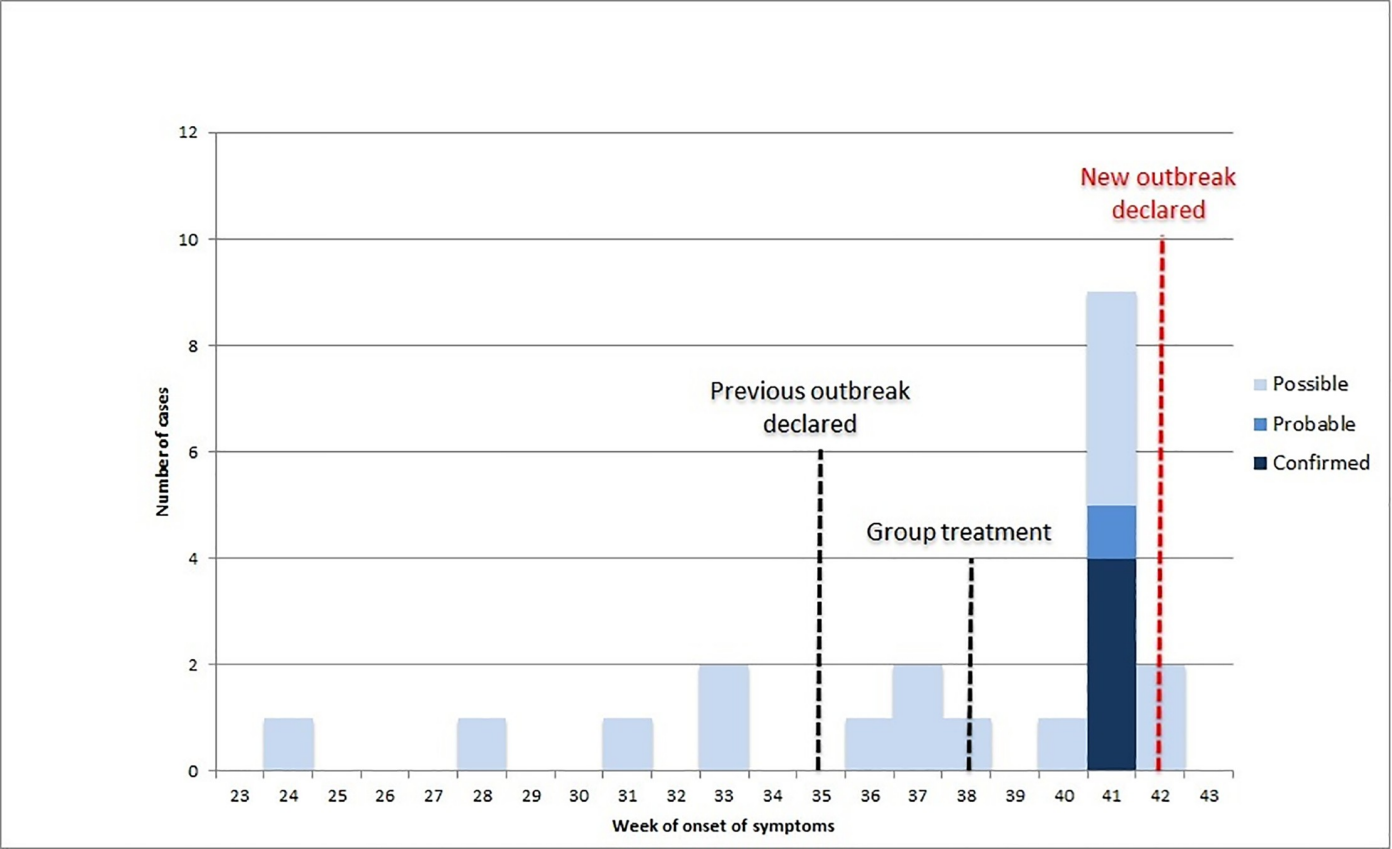
Epidemic curve of scabies cases among staff members and residents. The date of onset was available for 21 out of the 40 cases.

The epidemiological curve for the date of onset of illness (i.e. the date of onset of reported itchiness or a skin condition) for the residents and staff members is depicted in **Fig 1**. For 53% (21/40) of the cases there was a well-defined date of onset. The peak of the cases occurred in week 41; the earliest date of onset was in week 24. This outbreak occurred in the context of multiple scabies outbreaks in this nursing home; the last outbreak was declared in week 35 and group treatment was administered in week 38.

21 skin specimens were obtained: 15 from residents and 7 from staff members. From these skin specimens, three cases (2 residents, 1 staff) were confirmed by microscopy and one showed positive findings with both techniques. Three bed specimens of the residents were tested by microscopy and PCR. Among residents, the diagnosis for two additional cases of scabies (of in total six confirmed cases) was confirmed via PCR on skin flakes from bed linens only. These cases did not report symptoms or have typical lesions suggestive of scabies, and therefore no skin scrapings were taken.

## Discussion

We conducted an outbreak investigation of a large outbreak of scabies in a nursing home. We were able to confirm two additional cases by PCR performed on skin samples found on bed linens. To our knowledge, PCR on bed linens has not been used in an outbreak prior to this investigation. This technique could be beneficial for case finding which leads to more information on the true extent of the outbreak; therefore resulting in more efficient control measures to stop transmission of scabies. This is especially relevant in similar settings as this outbreak, where there is a vulnerable population of patients who cannot express whether they have symptoms suggestive of scabies. Of note: environmental cross-contamination was assumed unlikely as hygiene protocols were in place and had been audited several times during the series of outbreaks.

Strength of this study was that all the residents and a majority of the staff members were included, which made it possible to look at the full extent of the outbreak of scabies within this nursing home. Yet a limitation of this study was the limited information on disease onset; due to the residents’ psychiatric condition we had to rely on medical records to gather data.

**Fig 2:**
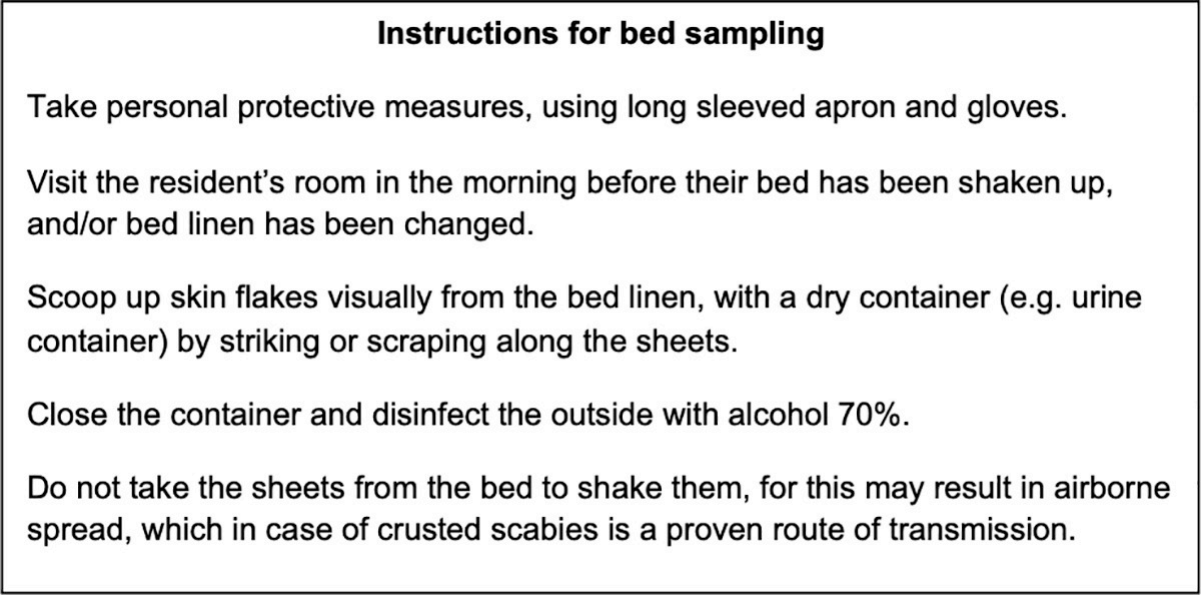
Instructions for bed sampling.

During this outbreak we examined nearly all the residents, leading us to detect additional cases of scabies in a group of residents that did not report symptoms prior to the investigation. However the physical examination of all persons during an outbreak can be a challenging logistical and financial task for any nursing home. Moreover this could also lead to mental anguish for this vulnerable population. Therefore a less complicated alternative could be to perform PCR on skin samples found on bed linens. In **Fig 2**, we described our best practices for collecting bed specimens.

In the course of this outbreak investigation control measures were advised. All residents and staff members were treated with a topical skin ointment (i.e. permetrin (Loxazol) 5%) and an oral drug (i.e. ivermectin (Stromectol) in a dosage of 3 mg per kilogram bodyweight). This treatment was repeated one week after the first treatment. This drug treatment protocol was instructed and advised in combination with standard hygiene measures for scabies, including environmental decontamination. All household contacts of infested staff members of the nursing home were also treated once with either a topical skin ointment or an oral drug, in combination with hygiene measures.

In the Netherlands every outbreak of scabies in a nursing home is monitored for 20 weeks; this is two times the maximum incubation period for patients living in care institutions. If any new cases are found within this period, the outbreak investigation is restarted and additional control measure could be advised. During this monitoring period no new cases of scabies were identified in this cohort of residents and staff members.

In conclusion, we recommend further evaluating the potential of scabies PCR on bed linens to contribute to case finding in outbreaks of scabies in institutions with vulnerable populations. Additional studies comparing the scabies PCR on bed linen specimens with PCR on skin scrapings from lesions are needed.
